# Solitary Calcified Nodules as the Cause of Carpal Tunnel Syndrome: Two Case Reports and Literature Reviews

**DOI:** 10.3389/fneur.2019.00224

**Published:** 2019-03-15

**Authors:** Ting-Feng Cheng, Chun-Yu Chen, Ping-Tang Liu, Shan-Wei Yang

**Affiliations:** ^1^Department of Orthopaedics, Kaohsiung Veterans General Hospital, Kaohsiung, Taiwan; ^2^Department of Orthopaedic Surgery, National Defense Medical Center, Taipei, Taiwan; ^3^Department of Occupational Therapy, Shu-Zen Junior College of Medicine and Management, Kaohsiung, Taiwan; ^4^Xiang Yang Rehabilitation Clinic, Kaohsiung, Taiwan

**Keywords:** carpal tunnel syndrome, calcified nodule, ultrasound, space-occupying lesion, secondary CTS

## Abstract

Solitary calcified nodule-related carpal tunnel syndrome (CTS) is rare and easy to be misdiagnosed owing to the high incidence of primary CTS. Release of the transverse carpal ligament without removal of the mass leads to persistence of the symptoms and subsequent complications like wasting of the thenar muscles. Here, we report two cases of solitary calcified nodule-related CTS and discuss the role of ultrasound in preventing misdiagnosis. Both patients reported persistent numbness over the lateral side of their palm and weakness of the right wrist with thenar muscle atrophy. One patient had undergone transverse carpal ligament release 2 years previously, and the other had received a local injection of lidocaine at the clinic. Neither patient experienced symptom relief. X-ray revealed a similar finding of nodule lesions in front of the capitate–hamate region. Solitary calcified nodule-related CTS was diagnosed, and the patients underwent nodule removal with/without transverse ligament release. The first patient was a typical case of misdiagnosed solitary calcified nodule-related CTS. The second patient had a definitive clinical sonographic diagnosis before surgery. The accurate diagnosis of secondary CTS is paramount for performing effective surgery. Thus, ultrasonography is an easy, convenient, safe, and effective method in screening for secondary CTS.

## Background

Secondary carpal tunnel syndrome (CTS) is less common than primary CTS wherein the median nerve is entrapped within the transverse carpal ligament. The pathological causes of secondary CTS may include trauma, dialysis-related amyloidosis, pregnancy, or space-occupying lesions (1). Space-occupying lesions can cause increasing pressure due to decreased space and increased content within the carpal tunnel. The common pathogeneses of space-occupying lesions include tenosynovitis, vascular anomalies, malunited distal radial fractures, pseudogout, gout arthritis, and tumoral calcinosis (2–4). Tumoral calcinosis, which presents as a solitary calcified nodule between the flexor tendon and the carpal bone, is a rare etiology of secondary CTS (5, 6). The symptoms of CTS may have continued because transverse carpal ligament release was performed without tumor removal owing to the failure to recognize the space-occupying lesion. Here, we report two cases with solitary calcified nodule-related CTS and present a review of the literature.

## Case Presentation

**Figure 1:**
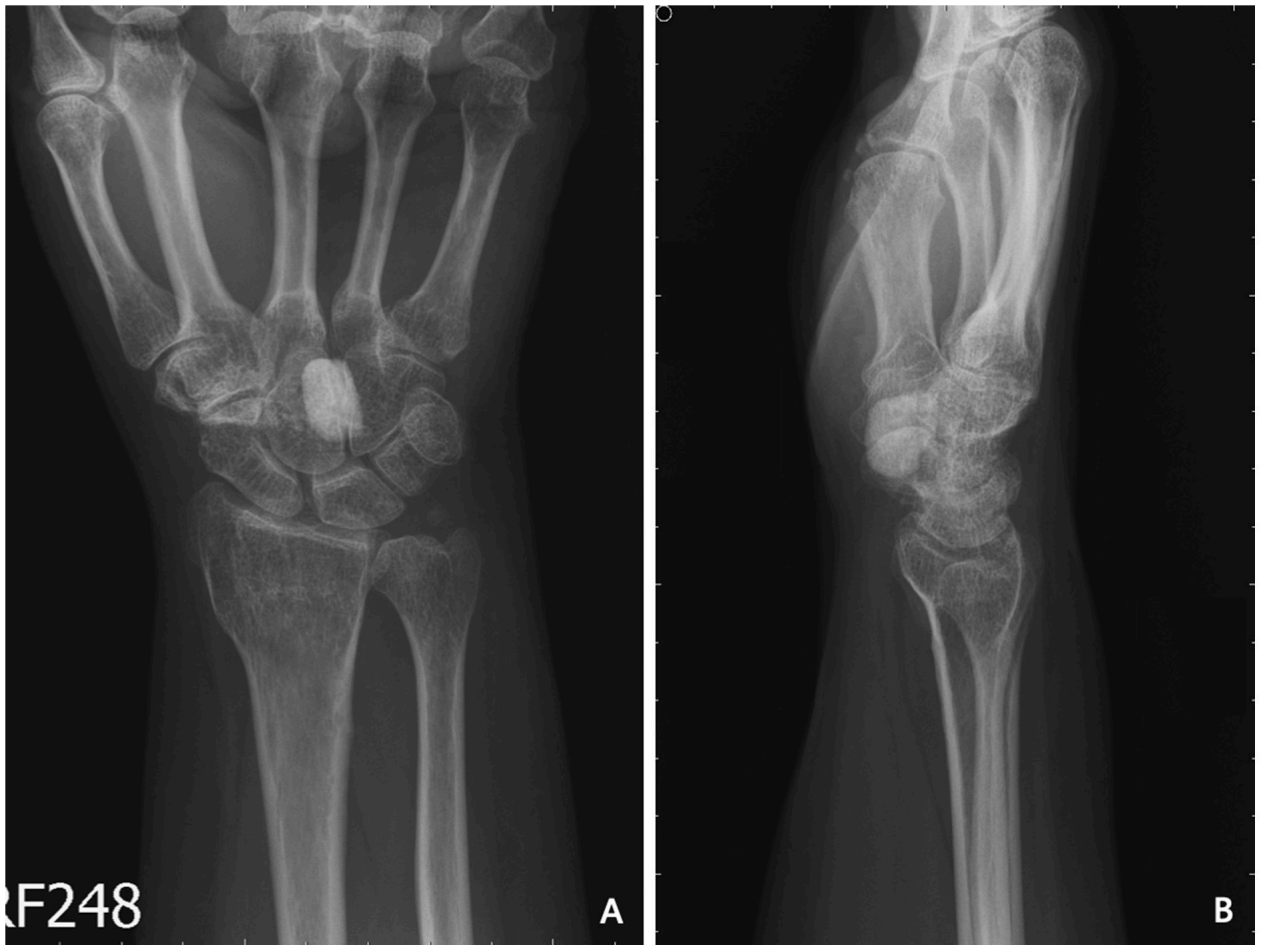
Preoperative X-ray of the first patient showing one calcified nodule sized 1.3 × 0.8 × 1.0 cm^3^ at the volar side of the capitate–hamate region. **(A)** Anteroposterior view. **(B)** Lateral view.

**Figure 2:**
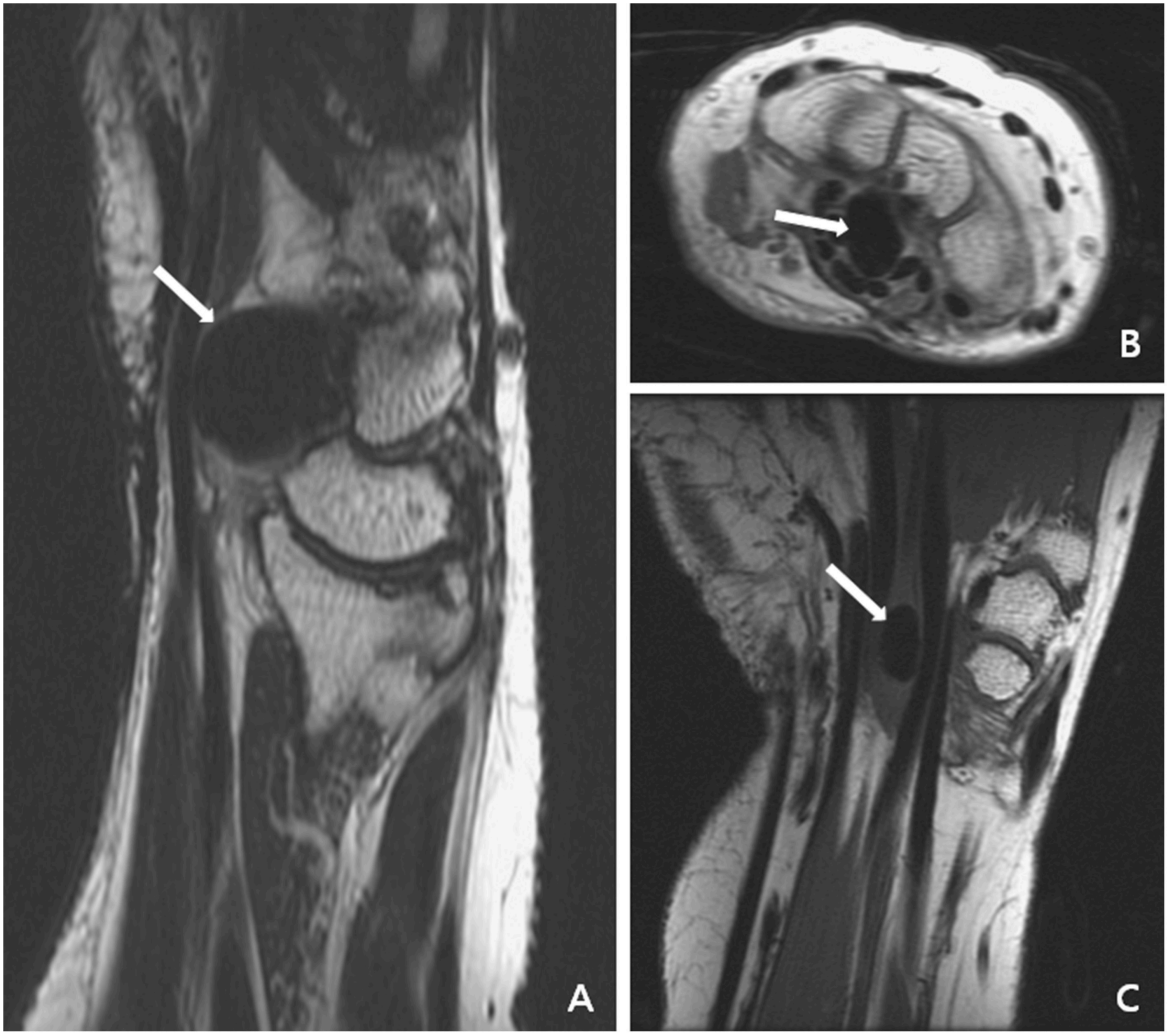
Magnetic resonance imaging of the first patient disclosed focal lower intensity of the nodular lesion (white arrow) without obvious contrast enhancement. **(A)** Sagittal plane of T2- and T1-weighted images. **(B)** Axial plane of T1-weighted image with contrast. **(C)** Coronal plane of T1-weighted image.

Our first patient was a 57-year-old female who presented to our outpatient department with complaints of persistent numbness over the lateral side of her palm and poor grip strength of her right hand after undergoing carpal tunnel release at another clinic 2 years previously. Physical examination revealed atrophy of the right thenar muscle and positive Tinel's sign and Phalen's test. X-ray revealed a solitary calcified nodule sized 1.3 × 0.8 × 1.0 cm^3^ at the volar side of the capitate–hamate region (Figure 1). Both T1- and T2-weighted magnetic resonance imaging revealed lower focal intensity of the nodular lesion without obvious contrast enhancement (Figure 2). A nodular lesion mimicking CTS was diagnosed, and the patient subsequently underwent tumor excision. Intraoperative findings revealed a solitary, whitish, well-margined nodule with easily crumbled content.

**Figure 3:**
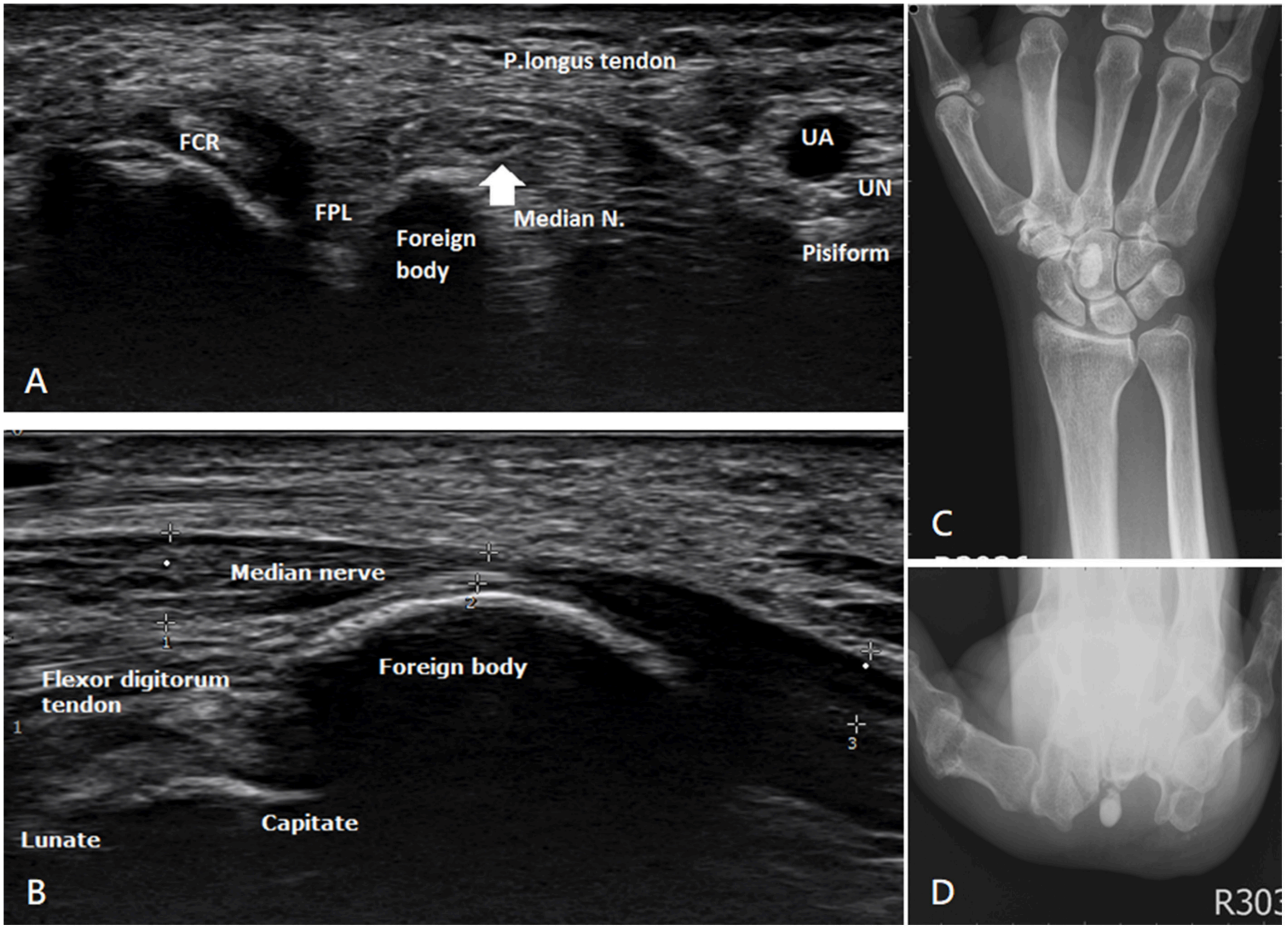
Ultrasonography and preoperative X-ray of the second patient. **(A)** Transverse view and **(B)** longitudinal view present one hyperechoic ovoid lesion occupying the carpal tunnel with obvious median nerve compression. **(C)** Anteroposterior view and **(D)** carpal tunnel view show one radiopaque nodule sized 0.6 × 0.6 × 1.3 cm^3^ in front of the capitate.

**Figure 4:**
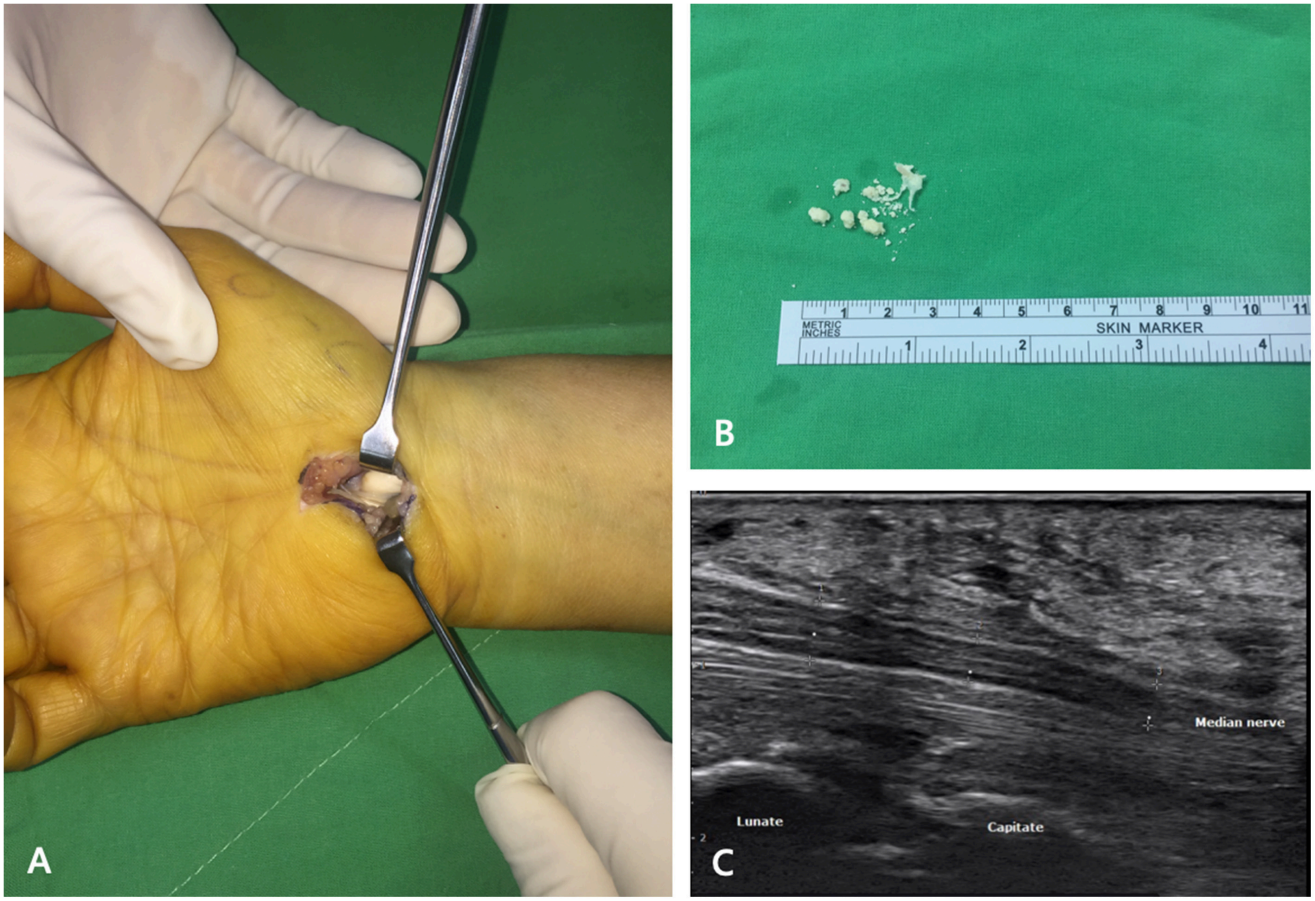
Intraoperative findings and postoperative sonography of the second patient. **(A)** Transverse carpal ligament was released and one whitish, well-margined nodule was observed over the carpal tunnel. **(B)** Grayish calcified nodule with easily crumbled content. **(C)** Postoperative sonography revealed no compression of the median nerve by foreign bodies.

Our second patient was a 52-year-old female with a history of medically controlled type II diabetes mellitus for 5 years. She reported numbness in her first, second, and third fingers with decreased grip strength of her right hand since 10 months ago. Physical examination revealed right hand thenar muscle atrophy and Tinel's sign and positive Phalen's test. She received a local injection of lidocaine at the clinic; however, her numbness did not improve as expected. Ultrasound was used as the initial imaging modality; the median nerve was scanned by the upper limbs nerve tracking protocol (7). The patient was made to lie on the bed in the supine position and was asked to maintain forearm supination. The ultrasound transducer was placed at middle of the patient's forearm, a position where the median nerve travels between the flexor digitorum superficialis and profundus tendons. Next, the transducer was moved to the distal area, where the median nerve passed from below the flexor retinaculum and tendons. The palmar cutaneous branch of median nerve (PCMN), which emerged from the radial aspect of the median nerve and circled around the upper border of the median nerve to reach the antebrachial fascia, was traced; PCMN passed through the antebrachial fascia and entered the ulnar side of the flexor carpi radialis tendon (8). A hyperechoic ovoid lesion with posterior shadowing between the median nerve and capitate, which led to compression of the median nerve, was noted (Figures 3A,B). X-ray also revealed one radiopaque nodule sized size of 0.6 × 0.6 × 1.3 cm^3^ in front of the capitate (Figures 3C,D). The patient was then referred to our outpatient department. Electromyography (EMG)/nerve conduction velocity (NCV) testing revealed moderate demyelination of the median nerve in the right hand. Resection biopsy and transverse carpal ligament release were performed; the intraoperative findings revealed a solitary, whitish, well-margined nodule over the carpal tunnel (Figures 4A,B). Postoperative sonography revealed no compression of the median nerve by foreign bodies (Figure 4C). The pathological findings of both patients indicate a grayish, calcified nodule. Both patients reported immediate improvements in their symptoms after surgery.

## Discussion

CTS is thought to be a common median nerve mononeuropathy; its characteristics include pain and numbness from the volar aspect of the thumb to the radial half of the ring finger. To the best of our knowledge, CTS has several pathogeneses, the most common being idiopathic. Other pathogeneses include local disease, such as inflammation, trauma, tumors, or anatomical anomalies, as well as regional etiology, including rheumatoid arthritis, gout, and osteoarthritis. Furthermore, some systemic causes, such as diabetes mellitus and pregnancy, can also cause CTS (1, 3).

Of all the above-mentioned etiologies, space-occupying lesions are the rarest; a retrospective study conducted routine radiography of the anteroposterior, lateral, and carpal tunnel views for the diagnosis of CTS and reported a relatively low incidence (3.4%) after reviewing 324 cases of CTS (9). Similarly, another retrospective study reported a 2.9% incidence of space-occupying lesions—only 23 out of 779 patients who underwent open carpal tunnel release had a lesion (10). In terms of the unusual incidence of space-occupying lesion-related CTS, not all surgeons may perform routine imaging owing to additional costs and uncertain reliability. Furthermore, this clinical status contributes to misdiagnoses and failure to perform adequate treatment. If secondary CTS cannot be distinguished from primary CTS, the most common clinical condition is similar to that of our first presenting case. Two years ago, our first patient had been misdiagnosed with primary CTS of the right hand rather than with space-occupying lesion-related CTS. Therefore, the numbness in her right hand was not relieved even after undergoing transverse carpal ligament release without nodule removal. Thus, deciding when to perform additional imaging to correct a possible misdiagnosis for space-occupying lesion-related CTS becomes an important issue.

A careful physical examination should be initially performed for establishing an accurate diagnosis. Identification of a space-occupying lesion becomes easy when the nodule is palpable and presents as swelling and local tenderness over the flexion crease of the wrist (9). The incidence of patients with unilateral CTS is higher than that of patients with bilateral CTS. A retrospective study (9) conducted in 2009 accumulated 324 cases of CTS and conducted routine radiographs for a survey; the results revealed a higher incidence of patients with unilateral CTS (12.5%) than of those with bilateral CTS (0.4%). Likewise, another retrospective study reviewed 128 cases of CTS and divided them into three groups: bilateral, subclinical, and unilateral CTS; the study reported no space-occupying lesion-related CTS in the bilateral or subclinical CTS groups; however, a significantly higher incidence of space-occupying lesion was noted in the unilateral CTS group (2).

Our second patient responded poorly to a local injection of lidocaine and exhibited typical symptoms of CTS during physical examination of the unilateral wrist. Due to obscure local tenderness and the small size, the lesion was difficult to locate via only physical examination. A hyperechoic ovoid lesion occupying the carpal tunnel and directly compressing the median nerve was detected using sonography. A subsequent X-ray also revealed a radiopaque nodule sized 0.6 × 0.6 × 1.3 cm^3^ in front of the capitate. Owing to the accurate diagnosis of space-occupying lesion-related CTS, the patient underwent surgery that included nodule excision and transverse carpal ligament release and achieved satisfying outcomes. All of the above-mentioned studies concluded that the possibility of space-occupying lesions should be considered when treating patients with typical symptoms of CTS at the initial presentation.

In both patients, the final pathological findings indicated a calcified nodule. Namba et al. inferred that the pathogenesis of calcified nodules in the carpal tunnel and calcifying tendinitis of the rotator cuff may be the same (5). They collected seven cases of calcified nodule-related CTS and analyzed the location and content of the calcified nodule using infrared absorption spectrometry. The calcified nodule was located over the area in front of the capitate–hamate region at the insertion of the volar radiocarpal extrinsic ligament. An inflammatory reaction would promote calcified nodule formation after repeated ligament pulling. The results of content analysis indicated the presence of 60% basic calcium phosphate, a finding similar to that observed in calcifying tendinitis. A calcified deposit with surrounding fibrocartilaginous tissue, as revealed by histological examination, also supports this evidence. No recurrent cases were found upon performing follow-up radiography in this study within 24 months (5).

With respect to the examination, electrodiagnosis, which can help evaluate the severity of mononeuropathy of the median nerve as well as establish a differential diagnosis, is the gold standard for diagnosis. Several drawbacks of the NCV/EMG tests have been noted, for example, it is an invasive and uncomfortable procedure and is associated with increasing costs and relatively low sensitivity (11). For the initial analysis of space-occupying lesion-related CTS, we suggest X-ray and sonography as the most suitable diagnostic tests owing to their accessibility. X-ray of the anteroposterior and lateral view as well as the carpal tunnel view is a useful and simple examination to identify a calcified lesion. However, sonography can detect not only radiopaque lesions but also radiolucent lesions, such as ganglion cysts, tenosynovitis, and vascular anomalies. Based on previous studies, ultrasonography has already been proven to relieve neuromuscular disease by measuring the quality and quantity of the muscle as well as the nerve cross-sectional area (CSA) (12, 13). Besides, the evaluation performed after intervention using static and dynamic ultrasonography imaging is also helpful (14). A prospective cross-sectional study used a sonographic parameter that includes the CSA of the median nerve at the carpal tunnel and mid-forearm to calculate the wrist-to-forearm ratio; the study reported that 26% of the patients with CTS had an abnormal CSA but normal or borderline nerve conduction studies (NCS). The results revealed a significant correlation between sonographic and electrodiagnostic studies; based on these results, sonography was suggested to be useful in the clinical diagnosis of patients with CTS with normal or borderline NCS (15).

As the initial imaging modality for secondary CTS, ultrasound is convenient, inexpensive, and non-invasive; further, it does not require additional radiation exposure and helps establish initial differential diagnoses in the outpatient department. When compared pre- and postoperatively, ultrasound can also be used to examine the decompression of the median nerve. Most importantly, ultrasound could prevent the misdiagnosis of space-occupying lesion-related CTS, thereby facilitating the performance of effective surgery.

## Ethics Statement

Written informed consent was obtained from the patient for the publication of this case report.

## Author Contributions

T-FC, C-YC, and S-WY contributed to the operation and writing. P-TL performed the ultrasound. All authors contributed to the manuscript revision and approved the submitted version.

### Conflict of Interest Statement

The authors declare that the research was conducted in the absence of any commercial or financial relationships that could be construed as a potential conflict of interest.
